# Supplementary material for: COVID-19 pandemic and food security in different contexts: A systematic review protocol
Source: PLoS One. 2022 Sep 12;17(9):e0273046. doi: 10.1371/journal.pone.0273046 (PMC9467351; doi:10.1371/journal.pone.0273046)
Supplement: S1 File — (DOCX) [file pone.0273046.s001.docx]

**MEDLINE search strategy (via PubMed)**

| **Database** | **Search terms** | **Item found** |
| --- | --- | --- |
| **Food Safety & Coronavirus** | | |
| **#1** | "food*"[Title] OR "beverag*"[Title] OR "fruit*"[Title] OR "vegetab*"[Title] OR "fresh*"[Title] OR "drink*"[Title] OR "juice*"[Title] OR "poult*"[Title] OR "meat*"[Title] OR "bread*"[Title] OR "bak"[Title] OR "cereal*"[Title] OR "dairy"[Title] OR "nurish*"[Title] OR "eat"[Title] OR "diet*"[Title] OR "feed*"[Title] OR "malnut*"[Title] | **598,928** |
| **#2** | "covid*"[Title/Abstract] OR "coronavir*"[Title/Abstract] OR "sars"[Title/Abstract] | **135,351** |
| **#3** | "safety"[Title/Abstract] OR "secur*"[Title/Abstract] OR "insecur*"[Title/Abstract] OR "povert*"[Title/Abstract] OR "sufficen*"[Title/Abstract] OR "insuffic*"[Title/Abstract] OR "risk*"[Title/Abstract] OR "uncertain*"[Title/Abstract] OR "hygien*"[Title/Abstract] OR "affluen*"[Title/Abstract] OR "suppl*"[Title/Abstract] OR "reserve*"[Title/Abstract] OR "avail*"[Title/Abstract] OR "access*"[Title/Abstract] OR "stabil*"[Title/Abstract] OR "utilize*"[Title/Abstract] | **6,010,563** |
| #4 | #1 AND #2 AND #3 | **557** |
| #4 | ("food*"[Title] OR "beverag*"[Title] OR "fruit*"[Title] OR "vegetab*"[Title] OR "fresh*"[Title] OR "drink*"[Title] OR "juice*"[Title] OR "poult*"[Title] OR "meat*"[Title] OR "bread*"[Title] OR "bak"[Title] OR "cereal*"[Title] OR "dairy"[Title] OR "nurish*"[Title] OR "eat"[Title] OR "diet*"[Title] OR "feed*"[Title] OR "malnut*"[Title]) AND ("covid*"[Title/Abstract] OR "coronavir*"[Title/Abstract] OR "sars"[Title/Abstract]) AND ("safety"[Title/Abstract] OR "secur*"[Title/Abstract] OR "insecur*"[Title/Abstract] OR "povert*"[Title/Abstract] OR "sufficen*"[Title/Abstract] OR "insuffic*"[Title/Abstract] OR "risk*"[Title/Abstract] OR "uncertain*"[Title/Abstract] OR "hygien*"[Title/Abstract] OR "affluen*"[Title/Abstract] OR "suppl*"[Title/Abstract] OR "reserve*"[Title/Abstract] OR "avail*"[Title/Abstract] OR "access*"[Title/Abstract] OR "stabil*"[Title/Abstract] OR "utilize*"[Title/Abstract]) | **557** |
| **ANOTHER SERACH** | | |
| #5 | "restaurant*"[Title/Abstract] OR "shop*"[Title/Abstract] OR "grocer*"[Title/Abstract] OR "cafe*"[Title/Abstract] OR "stuff*"[Title/Abstract] OR "court*"[Title/Abstract] | **60340** |
| #6 | #2 AND #5 | **506** |
| #6 | ("covid*"[Title/Abstract] OR "coronavir*"[Title/Abstract] OR "sars"[Title/Abstract]) AND ("restaurant*"[Title/Abstract] OR "shop*"[Title/Abstract] OR "grocer*"[Title/Abstract] OR "cafe*"[Title/Abstract] OR "stuff*"[Title/Abstract] OR "court*"[Title/Abstract]) | **506** |
|  | ANOTHER SEARCH |  |
| #7 | "cost*"[Title/Abstract] OR "price*"[Title/Abstract] OR "expendit*"[Title/Abstract] OR "income*"[Title/Abstract] OR "povert*"[Title/Abstract] OR "money"[Title/Abstract] | **873,862** |
| #8 | #1 AND #2 AND #7 | **150** |
| #8 | ("food*"[Title] OR "beverag*"[Title] OR "fruit*"[Title] OR "vegetab*"[Title] OR "fresh*"[Title] OR "drink*"[Title] OR "juice*"[Title] OR "poult*"[Title] OR "meat*"[Title] OR "bread*"[Title] OR "bak"[Title] OR "cereal*"[Title] OR "dairy"[Title] OR "nurish*"[Title] OR "eat"[Title] OR "diet*"[Title] OR "feed*"[Title] OR "malnut*"[Title]) AND ("covid*"[Title/Abstract] OR "coronavir*"[Title/Abstract] OR "sars"[Title/Abstract]) AND ("cost*"[Title/Abstract] OR "price*"[Title/Abstract] OR "expendit*"[Title/Abstract] OR "income*"[Title/Abstract] OR "povert*"[Title/Abstract] OR "money"[Title/Abstract] OR "race*"[Title/Abstract] OR "ethnic*"[Title/Abstract] OR "minorit*"[Title/Abstract] OR "discrim*"[Title/Abstract] OR " vulner*"[Title/Abstract]) | **150** |
| #9 | "wast*"[Title/Abstract] OR "stunt*"[Title/Abstract] OR "underweight"[Title/Abstract] OR "weight*"[Title/Abstract] OR "an em*"[Title/Abstract] OR "vitamin*"[Title/Abstract] OR "Protein*"[Title/Abstract] | 1,481,131 |
| #10 | #9 AND #2 AND #1 | **152** |
|  | ("wast*"[Title/Abstract] OR "stunt*"[Title/Abstract] OR "underweight"[Title/Abstract] OR "weight*"[Title/Abstract] OR "an em*"[Title/Abstract] OR "vitamin*"[Title/Abstract]) OR "Protein*"[Title/Abstract] AND ("covid*"[Title/Abstract] OR "coronavir*"[Title/Abstract] OR "sars"[Title/Abstract]) AND ("food*"[Title] OR "beverag*"[Title] OR "fruit*"[Title] OR "vegetab*"[Title] OR "fresh*"[Title] OR "drink*"[Title] OR "juice*"[Title] OR "poult*"[Title] OR "meat*"[Title] OR "bread*"[Title] OR "bak"[Title] OR "cereal*"[Title] OR "dairy"[Title] OR "nurish*"[Title] OR "eat"[Title] OR "diet*"[Title] OR "feed*"[Title] OR "malnut*"[Title]) | **152** |
